# XBP1 maintains beta cell identity, represses beta-to-alpha cell transdifferentiation and protects against diabetic beta cell failure during metabolic stress in mice

**DOI:** 10.1007/s00125-022-05669-7

**Published:** 2022-03-22

**Authors:** Kailun Lee, Jeng Yie Chan, Cassandra Liang, Chi Kin Ip, Yan-Chuan Shi, Herbert Herzog, William E. Hughes, Mohammed Bensellam, Viviane Delghingaro-Augusto, Mark E. Koina, Christopher J. Nolan, D. Ross Laybutt

**Affiliations:** 1 Garvan Institute of Medical Research, St Vincent’s Clinical School, UNSW Sydney, Darlinghurst, NSW Australia; 2 Secteur des sciences de la santé, Institut de recherche expérimentale et clinique, Pôle d’endocrinologie, diabète et nutrition, Université catholique de Louvain, Brussels, Belgium; 3 Medical School and John Curtin School of Medical Research, Australian National University, Canberra, ACT Australia; 4 ACT Pathology, Canberra Health Services, Garran, ACT Australia; 5 Department of Endocrinology, The Canberra Hospital, Garran, ACT Australia

**Keywords:** Beta cell identity, Dedifferentiation, Endoplasmic reticulum stress, Islets, Type 2 diabetes, Unfolded protein response

## Abstract

**Aims/hypothesis:**

Pancreatic beta cell dedifferentiation, transdifferentiation into other islet cells and apoptosis have been implicated in beta cell failure in type 2 diabetes, although the mechanisms are poorly defined. The endoplasmic reticulum stress response factor X-box binding protein 1 (XBP1) is a major regulator of the unfolded protein response. XBP1 expression is reduced in islets of people with type 2 diabetes, but its role in adult differentiated beta cells is unclear. Here, we assessed the effects of *Xbp1* deletion in adult beta cells and tested whether XBP1-mediated unfolded protein response makes a necessary contribution to beta cell compensation in insulin resistance states.

**Methods:**

Mice with inducible beta cell-specific *Xbp1* deletion were studied under normal (chow diet) or metabolic stress (high-fat diet or obesity) conditions. Glucose tolerance, insulin secretion, islet gene expression, alpha cell mass, beta cell mass and apoptosis were assessed. Lineage tracing was used to determine beta cell fate.

**Results:**

Deletion of *Xbp1* in adult mouse beta cells led to beta cell dedifferentiation, beta-to-alpha cell transdifferentiation and increased alpha cell mass. Cell lineage-specific analyses revealed that *Xbp1* deletion deactivated beta cell identity genes (insulin, *Pdx1*, *Nkx6.1*, *Beta2*, *Foxo1*) and derepressed beta cell dedifferentiation (*Aldh1a3*) and alpha cell (glucagon, *Arx*, *Irx2*) genes. *Xbp1* deletion in beta cells of obese *ob/ob* or high-fat diet-fed mice triggered diabetes and worsened glucose intolerance by disrupting insulin secretory capacity. Furthermore, *Xbp1* deletion increased beta cell apoptosis under metabolic stress conditions by attenuating the antioxidant response.

**Conclusions/interpretation:**

These findings indicate that XBP1 maintains beta cell identity, represses beta-to-alpha cell transdifferentiation and is required for beta cell compensation and prevention of diabetes in insulin resistance states.

**Graphical abstract:**

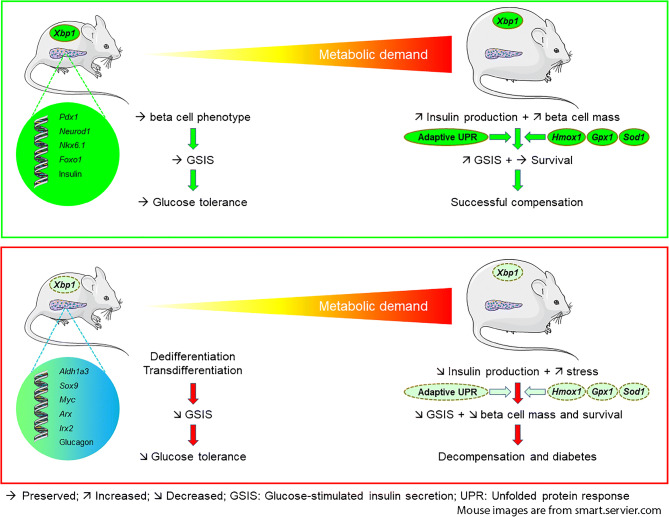

**Supplementary Information:**

The online version of this article 10.1007/s00125-022-05669-7 contains peer-reviewed but unedited supplementary material.

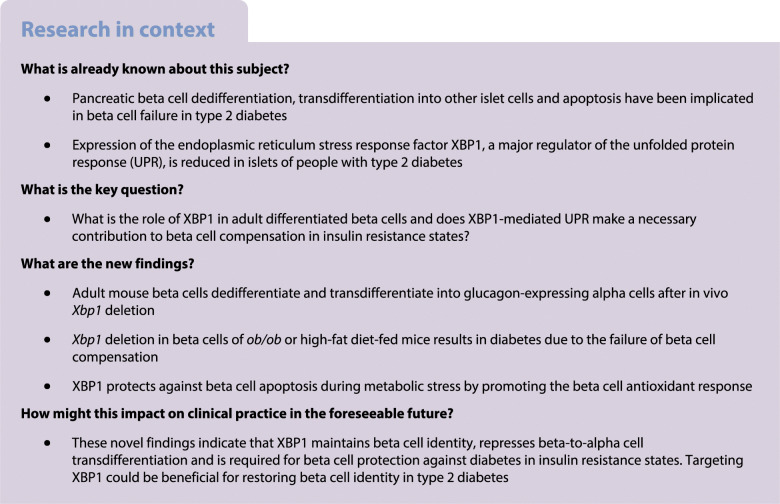

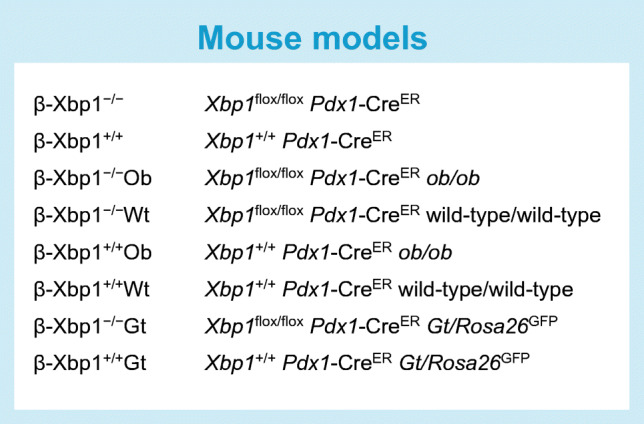





## Introduction

The ability of pancreatic beta cells to augment insulin production and secretion in response to increased metabolic demand is crucial for the maintenance of normoglycaemia. This adaptive response, termed beta cell compensation, is associated with both enhanced beta cell function and expansion of beta cell mass [1–3]. Hyperglycaemia develops when the demand for insulin exceeds the functional capacity of beta cells. Beta cell dysfunction occurs early in the aetiology of type 2 diabetes and deteriorates as the diabetic state worsens and beta cell capacity declines [4]. Deficits in adaptive beta cell mass linked with increased apoptosis are thought to contribute to the course of type 2 diabetes [5]. While much attention has focused on elucidating the mechanisms of beta cell compensation and failure in the development and progression of type 2 diabetes, they remain poorly understood.

Beta cell dedifferentiation has been proposed as an important contributor to beta cell failure in type 2 diabetes [6–9]. Evidence from animal and human studies suggests that mature beta cells lose their differentiated phenotype and cellular identity and regress to a less differentiated state and/or transdifferentiate into other islet cells in type 2 diabetes. While a major role of glucotoxicity in beta cell dedifferentiation has been established [6, 7, 10–12], the precise molecular mechanisms involved remain unclear.

The production of large quantities of insulin in beta cells imposes significant burden on the endoplasmic reticulum (ER) [13, 14]. Adaptation to ER stress by unfolded protein response (UPR) activation is proposed to play a protective role in increased insulin production, whereas the failure of adaptation has been implicated in beta cell dysfunction and apoptosis [15–20]. The UPR is mediated by three transmembrane stress sensor proteins: PKR-like ER kinase (PERK), inositol-requiring enzyme 1 (IRE1) and activating transcription factor 6 (ATF6). IRE1 cleaves the *Xbp1* mRNA, generating a transcription factor that promotes ER biogenesis and activates the expression of ER chaperone genes that are required for the folding and trafficking of secretory proteins. X-box binding protein 1 (XBP1) is an important regulator of the ER stress response in beta cells [18, 21–28]. Decreased spliced XBP1 expression was observed in islets of type 2 diabetic patients [29] and studies adopting embryonic deletion of *Xbp1* using the rat insulin promoter showed that it is required for optimal beta cell development and proinsulin processing [21]. Here, we employed inducible deletion of *Xbp1* specifically in beta cells of adult mice to investigate the function of XBP1 in the adaptive response of mature beta cells to increased metabolic demand.

## Methods

For detailed methods, please refer to the electronic supplementary material (ESM) Methods.

### Mouse models

To enable tamoxifen-inducible beta cell-specific deletion of *Xbp1*, we crossed *Pdx1*-Cre^ER^ mice [Tg(*Pdx1*-cre/Esr1*)#Dam/J, The Jackson Laboratory, Bar Harbour, ME, USA] with *Xbp1*^flox^ mice kindly provided by L. Glimcher, Dana-Farber Cancer Institute, Harvard University, Boston, MA, USA [21]. Experimental mice received i.p. tamoxifen (Sigma-Aldrich, St Louis, MO, USA) injections (3 × 75 mg/kg body weight) at 8–10 weeks of age to generate *Xbp1*^flox/flox^ *Pdx1*-Cre^ER^ (denoted β-Xbp1^−/−^) and littermate control *Xbp1*^+/+^ *Pdx1*-Cre^ER^ (β-Xbp1^+/+^) mice (ESM Fig. 1). Male littermates were assigned randomly to either chow or a high-fat diet.

To enable tamoxifen-inducible beta cell-specific deletion of *Xbp1* in obese mice, we crossed *Xbp1*^flox/flox^ *Pdx1*-Cre^ER^ mice with *ob*/+ (B6.Cg-*Lep*^*ob*^/J; The Jackson Laboratory) mice. Experimental mice received i.p. tamoxifen injections at 8–10 weeks of age to generate *Xbp1*^flox/flox^ *Pdx1*-Cre^ER^ *ob/ob* (β-Xbp1^−/−^Ob) and *Xbp1*^flox/flox^ *Pdx1*-Cre^ER^ wild-type/wild-type (β-Xbp1^−/−^Wt) mice and control *Xbp1*^+/+^ *Pdx1*-Cre^ER^ *ob/ob* (β-Xbp1^+/+^Ob) and *Xbp1*^+/+^ *Pdx1*-Cre^ER^ wild-type/wild-type (β-Xbp1^+/+^Wt) mice.

A model for cell type-specific lineage tracing and transcript profiling was developed by crossing *Xbp1*^flox/flox^ *Pdx1*-Cre^ER^ mice with *Gt/Rosa26*^GFP^ [*Gt(ROSA)26Sor*^*t*m9(EGFP/Rpl10a)Amc^; The Jackson Laboratory] mice. Mice received i.p. tamoxifen injections at 8–10 weeks of age to generate *Xbp1*^flox/flox^ *Pdx1*-Cre^ER^ *Gt/Rosa26*^GFP^ (β-Xbp1^−/−^Gt) and control *Xbp1*^+/+^ *Pdx1*-Cre^ER^ *Gt/Rosa26*^GFP^ (β-Xbp1^+/+^Gt) mice (see text box for mouse models).

### Glucose tolerance test

Glucose tolerance tests were performed after a 6 h fast, by oral gavage administration of glucose (1.5 or 3 g/kg body weight).

### Islet isolation and insulin secretion assays

Islets were isolated by Liberase digestion (Roche Diagnostics, Castle Hill, Australia), gradient centrifugation (Ficoll-Paque PLUS gradient, GE Healthcare Bio-Sciences, Uppsala, Sweden) and handpicking under a stereomicroscope. To assess ex vivo insulin secretion, batches of five islets were incubated in KRB-HEPES supplemented with 0.1% BSA containing either 2 or 20 mmol/l glucose for 1 h at 37°C. Secreted insulin was determined using the Insulin Ultra-Sensitive Assay (Cisbio, Codolet, France). The islets were lysed for measurement of insulin and DNA content.

### Glucolipotoxicity and antioxidant treatment ex vivo

Islets isolated from littermate *Xbp1*^+/+^ *Pdx1*-Cre^ER^ and *Xbp1*^flox/flox^ *Pdx1*-Cre^ER^ mice were treated with 100 nmol/l 4-hydroxy tamoxifen (Sigma-Aldrich) to generate β-Xbp1^+/+^ and β-Xbp1^−/−^ islets. Next, islets were treated in islet media with either 0.92% BSA (control) or 25 mmol/l glucose and 0.4 mmol/l palmitate coupled to 0.92% BSA (termed glucose + palmitate, GP) for 72 h at 37°C. For antioxidant treatment experiments, islets were co-treated with 2.5 mmol/l *N*-acetyl-l-cysteine (NAC) (Sigma-Aldrich). Cell death was determined in islets using the Cell Death Detection ELISA^PLUS^ Kit (Roche Diagnostics).

### Histology and immuno-morphometry

Formalin-fixed paraffin-embedded pancreases were sectioned and immunostained using the antibodies indicated (ESM Table 1). Slides were imaged using a Leica DM5500 fluorescent microscope or Leica DM6000 Power Mosaic microscope (Leica Microsystems, Wetzlar, Germany). Immunostaining was quantified using ImageJ/FIJI (ImageJ Developers; version 1.52a; https://imageJ.nih.gov/ij/; and FIJI Contributors; version 2.0.0-rc-69/1.52n; https://fiji.sc/).

### Immunoblotting

Islets were analysed by immunoblotting for XBP1, 14–3-3, phosphorylated IRE1 and total IRE1 using the antibodies indicated (ESM Table 1).

### RNA analysis

Real-time PCR was performed on the 7900 HT Real Time PCR System (Applied Biosystems, Foster City, CA, USA) using oligonucleotide primers (ESM Table 2). The value obtained for each specific gene product was normalised to a housekeeping gene (cyclophilin A) and expressed as a fold change of the value in control extracts.

### Translating ribosome affinity purification

Translating ribosome affinity purification (TRAP) was performed based on the previously published protocol [30, 31].

### Statistical analysis

All data are represented as means ± SEM. Unpaired two-tailed *t* test was used to compare differences between two groups. Differences between more than two groups were calculated using two-way ANOVA with Tukey’s post hoc test.

## Results

### Beta cell-specific deletion of *Xbp1* in high-fat diet-fed mice triggers diabetes by disrupting beta cell capacity

**Fig. 1:**
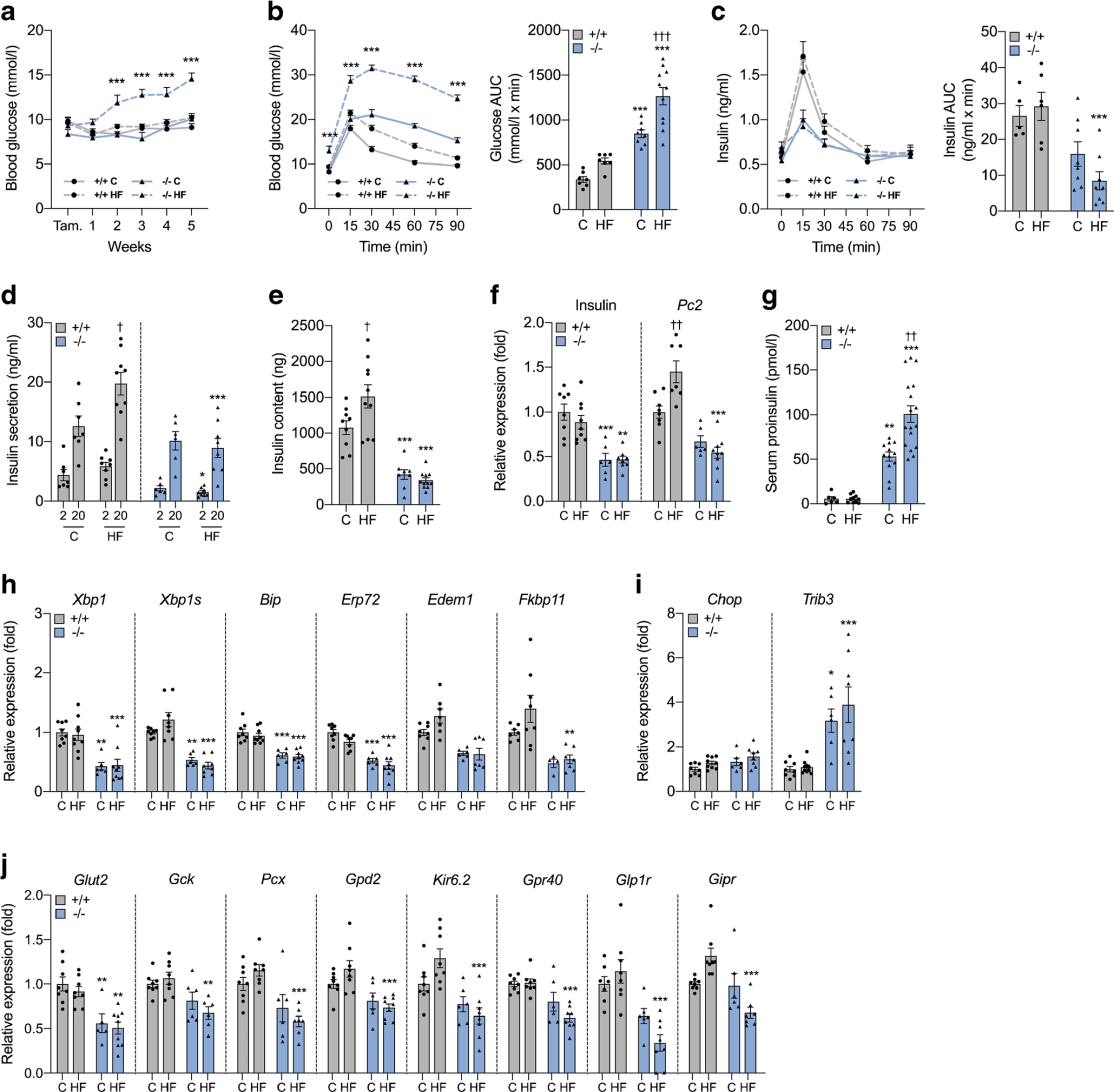
Beta cell-specific *Xbp1* deletion in adult mice fed a high-fat diet triggers diabetes by disrupting insulin secretory capacity. (**a**) Fed blood glucose levels. (**b**) Blood glucose levels and AUC for glucose during OGTT. (**c**) Serum insulin levels and AUC for insulin during OGTT. (**d**) Insulin secretion from isolated islets treated with low (2 mmol/l) or high (20 mmol/l) stimulatory level of glucose. (**e**) Insulin content in islets. (**f**) mRNA expression of insulin and *Pc2* in islets expressed as fold change of the levels in chow-fed β-Xbp1^+/+^ mice. (**g**) Serum proinsulin levels. (**h**) mRNA expression of adaptive UPR genes in islets expressed as fold change of the levels in chow-fed β-Xbp1^+/+^ mice. (**i**) mRNA expression of pro-apoptosis UPR genes in islets expressed as fold change of the levels in chow-fed β-Xbp1^+/+^ mice. (**j**) mRNA expression of beta cell function genes in islets expressed as fold change of the levels in chow-fed β-Xbp1^+/+^ mice. All data are represented as means ± SEM. *n* = 5–11, chow-fed β-Xbp1^+/+^; *n* = 6–19, high-fat-fed β-Xbp1^+/+^; *n* = 5–16, chow-fed β-Xbp1^−/−^; *n* = 6–18, high-fat-fed β-Xbp1^−/−^. ANOVA: *p<0.05, **p<0.01, ***p<0.001 genotype effect; ^†^*p*<0.05, ^††^*p*<0.01, ^†††^*p*<0.001 diet effect. C, chow-fed; HF, high-fat-fed; Tam., tamoxifen

Adult mice with specific *Xbp1* deletion in beta cells (*Xbp1*^flox/flox^ *Pdx1*-Cre^ER^, denoted β-Xbp1^−/−^) and littermate control mice (*Xbp1*^+/+^ *Pdx1*-Cre^ER^, denoted β-Xbp1^+/+^) were fed a chow or a high-fat diet for 5 weeks. Body weight was not different among the groups (ESM Fig. 2). Morning fed blood glucose levels in chow-fed β-Xbp1^−/−^ mice were not different from chow- and high-fat-fed β-Xbp1^+/+^ mice throughout the study (Fig. 1a). In contrast, the blood glucose levels in high-fat-fed β-Xbp1^−/−^ mice were significantly increased by 2 weeks after commencement of the diet and remained significantly higher compared with all the other groups throughout the study (Fig. 1a). The AUC for blood glucose levels from 0 to 90 min during OGTT was significantly increased in chow-fed β-Xbp1^−/−^ mice compared with β-Xbp1^+/+^ mice (Fig. 1b), indicating that the adult-onset deletion of *Xbp1* impairs glucose tolerance. After high-fat feeding, β-Xbp1^−/−^ mice displayed an elevated fasting glucose and severely worsened glucose tolerance compared with all other groups (Fig. 1b). These findings demonstrate that the deletion of *Xbp1* in beta cells of adult mice that are normally resistant to the development of diabetes induces glucose intolerance, with severe worsening and the development of diabetes when challenged by high-fat diet feeding.

The AUC for insulin levels during the OGTT was markedly reduced in high-fat-fed β-Xbp1^−/−^ mice compared with β-Xbp1^+/+^ mice (Fig. 1c). This shows that the deterioration of glucose tolerance in β-Xbp1^−/−^ mice after high-fat feeding was associated with lowered plasma insulin levels.

Compared with chow-fed mice, insulin secretion at a high stimulatory level of glucose (20 mmol/l) was increased in islets isolated from high-fat-fed β-Xbp1^+/+^ mice (Fig. 1d). Additionally, insulin content was increased in islets from high-fat-fed β-Xbp1^+/+^ mice compared with chow-fed controls (Fig. 1e). These findings are consistent with the usual enhancement of beta cell insulin secretory capacity in response to high-fat diet-induced insulin resistance. There were no differences detected in insulin secretion at both low and high stimulatory levels of glucose between islets from chow-fed β-Xbp1^+/+^ and β-Xbp1^−/−^ mice (Fig. 1d). However, after high-fat feeding, islets of β-Xbp1^−/−^ mice displayed significantly reduced insulin secretion at both low and high glucose levels compared with β-Xbp1^+/+^ mice (Fig. 1d). Furthermore, insulin content was reduced in islets from β-Xbp1^−/−^ mice, with no difference between the diets (Fig. 1e). This was associated with significantly reduced insulin mRNA levels in islets of β-Xbp1^−/−^ mice compared with β-Xbp1^+/+^ mice, with no effects of diet (Fig. 1f). Ultrastructure analysis by transmission electron microscopy (ESM Methods) revealed that some beta cells from β-Xbp1^−/−^ mice displayed fewer insulin granules compared with β-Xbp1^+/+^ mice, irrespective of diet (ESM Fig. 3). Serum proinsulin levels were increased in β-Xbp1^−/−^ mice fed a chow diet, and were increased further after high-fat feeding (Fig. 1g). The mRNA levels of the proinsulin processing enzyme, prohormone convertase-2 (*Pc2*, also known as *Pcsk2*), were increased in islets from β-Xbp1^+/+^ mice fed a high-fat diet, whereas they were reduced in islets from β-Xbp1^−/−^ mice after high-fat feeding (Fig. 1f). These data suggest that *Xbp1* deletion lowers islet insulin content, impairs proinsulin processing and disrupts the capacity of beta cells for insulin secretory adaptation in response to high-fat feeding, ultimately resulting in diabetes.

### XBP1 maintains expression of genes involved in the UPR and beta cell function

The expression of *Xbp1* and adaptive UPR genes, including ER chaperones, foldases and ER-associated degradation (ERAD) components [*Bip* (also known as *Hspa5*), *Erp72* (also known as *Pdia4*), *Edem1* and *Fkbp11*], and *Xbp1* splicing were reduced in islets from β-Xbp1^−/−^ mice compared with islets from β-Xbp1^+/+^ mice, with no differences between the diets (Fig. 1h). The expression of the pro-apoptotic UPR gene, *Chop* (also known as *Ddit3*), was not different between genotypes or diets, whereas *Trib3* expression was significantly increased in islets of β-Xbp1^−/−^ mice compared with β-Xbp1^+/+^ mice fed either a chow or high-fat diet (Fig. 1i).

We examined the expression of genes important for beta cell function. The glucose transporter, *Glut2* (also known as *Slc2a2*), was significantly downregulated in the islets of β-Xbp1^−/−^ mice compared with control β-Xbp1^+/+^ mice fed either a chow or a high-fat diet (Fig. 1j). However, the mRNA levels of the other beta cell function genes assessed [*Gck*, *Pcx*, *Gpd2*, *Kir6.2* (also known as *Kcnj11*), *Gpr40* (also known as *Ffar1*), *Glp1r* and *Gipr*] were reduced solely in islets of β-Xbp1^−/−^ mice fed a high-fat diet (Fig. 1j). These findings suggest that XBP1 is required to maintain the expression of genes important for beta cell function in mice challenged by a high-fat diet.

### *Xbp1* deletion alters islet cell composition and enhances beta cell turnover

**Fig. 2:**
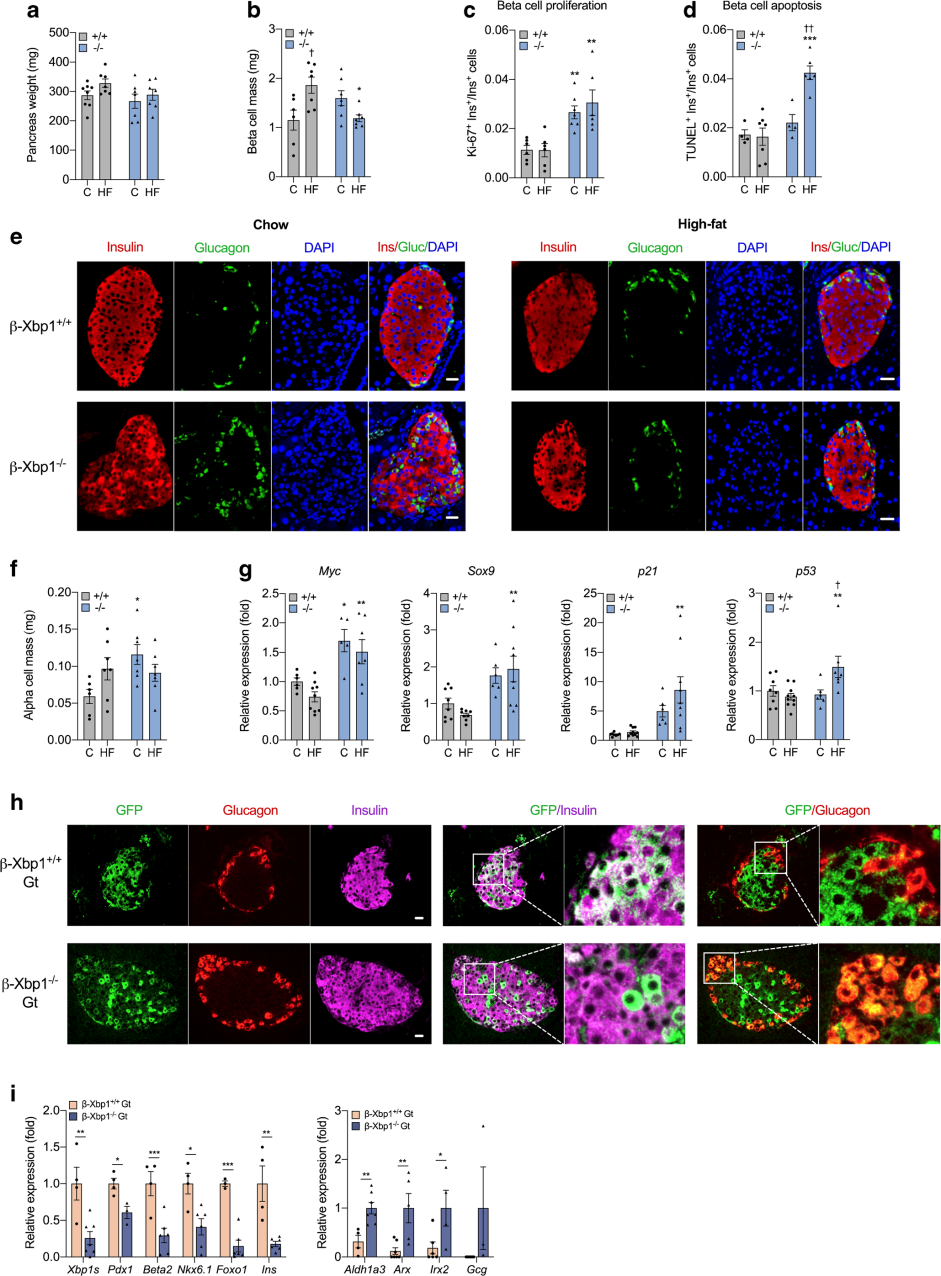
*Xbp1* deletion leads to altered islet cell composition, increased beta cell turnover, beta cell dedifferentiation and beta-to-alpha cell transdifferentiation. (**a**) Pancreas weight. (**b**) Beta cell mass (quantification of immunostaining for insulin). (**c**) Beta cell proliferation rate (quantification of immunostaining for Ki-67 and insulin). (**d**) Beta cell apoptosis rate (quantification of immunostaining for TUNEL and insulin). (**e**) Representative images of immunostaining for insulin and glucagon. (**f**) Alpha cell mass (quantification of immunostaining for glucagon). (**g**) mRNA expression of genes involved in beta cell proliferation (*Myc*), dedifferentiation (*Sox9*) and senescence (*p21* and *p53*) in islets, expressed as fold change of the levels in chow-fed β-Xbp1^+/+^ mice. (**h**) Representative images of immunostaining for GFP (green), glucagon (red) and insulin (magenta). Scale bar, 20 μm. (**i**) Expression in immunoprecipitated mRNA of beta cell identity (*Pdx1*, *Beta2*, *Nkx6.1* and *Foxo1*) genes expressed as fold change of the levels in β-Xbp1^+/+^Gt mice, and beta cell dedifferentiation (*Aldh1a3*) and alpha cell (*Arx*, *Irx2* and *Gcg*) genes expressed as fold change of the levels in β-Xbp1^−/−^Gt mice. All data are represented as means ± SEM. (**a**–**g**) *n* = 4–8, chow-fed β-Xbp1^+/+^; *n* = 6–10, high-fat-fed β-Xbp1^+/+^; *n* = 4–7, chow-fed β-Xbp1^−/−^; *n* = 6–9, high-fat-fed β-Xbp1^−/−^. ANOVA: *p<0.05, **p<0.01, ***p<0.001 genotype effect; ^†^*p*<0.05, ^††^*p*<0.01 diet effect. (**h**, **i**) *n* = 3–7, β-Xbp1^+/+^Gt; *n* = 3–8, β-Xbp1^−/−^Gt. Unpaired two-tailed *t* test: *p<0.05, **p<0.01, ***p<0.001. C, chow-fed; HF, high-fat-fed. Gluc, glucagon; Ins, insulin

Total pancreas weights were not different between β-Xbp1^+/+^ and β-Xbp1^−/−^ mice, irrespective of diet (Fig. 2a). Beta cell mass was significantly increased in high-fat-fed β-Xbp1^+/+^ mice compared with chow-fed controls (Fig. 2b). However, relative to high-fat-fed β-Xbp1^+/+^ mice, beta cell mass was reduced in high-fat-fed β-Xbp1^−/−^ mice, with values similar to those observed in chow-fed β-Xbp1^+/+^ mice (Fig. 2b). A non-significant increase in beta cell mass was apparent in chow-fed β-Xbp1^−/−^ mice compared with chow-fed controls (Fig. 2b). Interestingly, the rate of beta cell proliferation, assessed using Ki-67 immunostaining, was significantly increased in β-Xbp1^−/−^ mice compared with β-Xbp1^+/+^ mice, irrespective of diet (Fig. 2c). The rate of beta cell apoptosis, assessed using TUNEL staining, was unchanged in chow-fed β-Xbp1^−/−^ mice, but was significantly increased in β-Xbp1^−/−^ mice after high-fat feeding (Fig. 2d). These findings suggest that *Xbp1* deletion leads to beta cell proliferation and expansion under control conditions, but to high beta cell turnover after high-fat feeding, with the increase in beta cell proliferation outstripped by apoptosis, resulting in a relative decline of beta cell mass.

Whereas the β-Xbp1^+/+^ mice showed a typical organisation of mouse islets with a beta cell core and alpha cells distributed around the mantle, the islet pattern in β-Xbp1^−/−^ mice was often altered, with alpha cells distributed throughout the islets (Fig. 2e). Strikingly, alpha cell mass was increased by ~2-fold in β-Xbp1^−/−^ mice compared with β-Xbp1^+/+^ mice fed a chow diet, but was not different between the genotypes after high-fat feeding (Fig. 2f).

### *Xbp1* deletion increases expression of cell proliferation, senescence and inflammation markers

The expression of *Myc*, a cell cycle regulator [32], was increased in islets from β-Xbp1^−/−^ mice fed either a chow or a high-fat diet (Fig. 2g) in parallel with enhanced beta cell proliferation. Furthermore, the expression levels of the pancreatic progenitor and beta cell dedifferentiation marker, *Sox9* [33], cell cycle/senescence markers, *p21* (also known as *Cdkn1a*) and *p53* (also known as *Trp53*) (Fig. 2g), inflammatory cytokines (*Il6*, *Il1b* and *Tnf*) and the macrophage marker, *F4/80* (also known as *Adgre1*) (ESM Fig. 4), were significantly increased in islets from high-fat-fed β-Xbp1^−/−^ mice. Reduced insulin production can induce beta cell proliferation [14].

### *Xbp1* deletion leads to beta cell dedifferentiation and beta-to-alpha cell transdifferentiation

We next deleted *Xbp1* in adult beta cells and tracked their fate using a gene trap *Rosa26*^GFP^ lineage label in *Xbp1*^flox/flox^ *Pdx1*-Cre^ER^ *Gt/Rosa26*^GFP^ (β-Xbp1^−/−^Gt) and control *Xbp1*^+/+^ *Pdx1*-Cre^ER^ *Gt/Rosa26*^GFP^ (β-Xbp1^+/+^Gt) mice (ESM Fig. 5). Five weeks after tamoxifen administration, GFP staining was co-localised with insulin and not with glucagon in pancreases of β-Xbp1^+/+^Gt mice, as expected (Fig. 2h). However, in β-Xbp1^−/−^Gt mice, some cells stained for GFP but not for insulin, and substantial co-staining of GFP and glucagon was observed (Fig. 2h). This shows that deletion of *Xbp1* in adult beta cells leads to both a loss of insulin and the production of glucagon. The conversion of beta cells to glucagon-producing cells provides a mechanism for increased alpha cell mass in mice with *Xbp1* deletion.

We then used gene trap mice to conduct TRAP experiments. GFP^+^ ribosomal complexes were purified from islets isolated from chow-fed β-Xbp1^+/+^Gt and β-Xbp1^−/−^Gt mice in which the GFP–L10a ribosomal fusion protein is produced only in *Pdx1*-Cre^ER^-labelled beta cells and derived cells. Using quantitative PCR analysis, *Xbp1s* mRNA levels were shown to be significantly reduced in islets from β-Xbp1^−/−^Gt mice compared with control β-Xbp1^+/+^Gt mice (Fig. 2i). We then examined the expression of four transcription factors that are critical for the maintenance of beta cell identity in adult islets. Notably, *Pdx1*, *Beta2* (also known as *Neurod1*), *Nkx6.1* and *Foxo1* were significantly reduced in the islets of β-Xbp1^−/−^Gt mice compared with control mice (Fig. 2i). This was associated with an 82% reduction in insulin mRNA levels in β-Xbp1^−/−^Gt mice, whereas expression of the beta cell dedifferentiation marker *Aldh1a3* [8] was markedly increased (Fig. 2i). Next, we assessed the expression of genes restricted to alpha cells in adult animals. Strikingly, *Arx*, *Irx2* and glucagon *(Gcg)* mRNA levels were markedly increased in β-Xbp1^−/−^Gt mice compared with control mice (Fig. 2i). This suggests that *Xbp1* deletion in adult beta cells leads to beta-to-alpha cell reprogramming.

### *Xbp1* deletion in beta cells of obese o*b/ob* mice leads to failure of beta cell compensation and diabetes

**Fig. 3:**
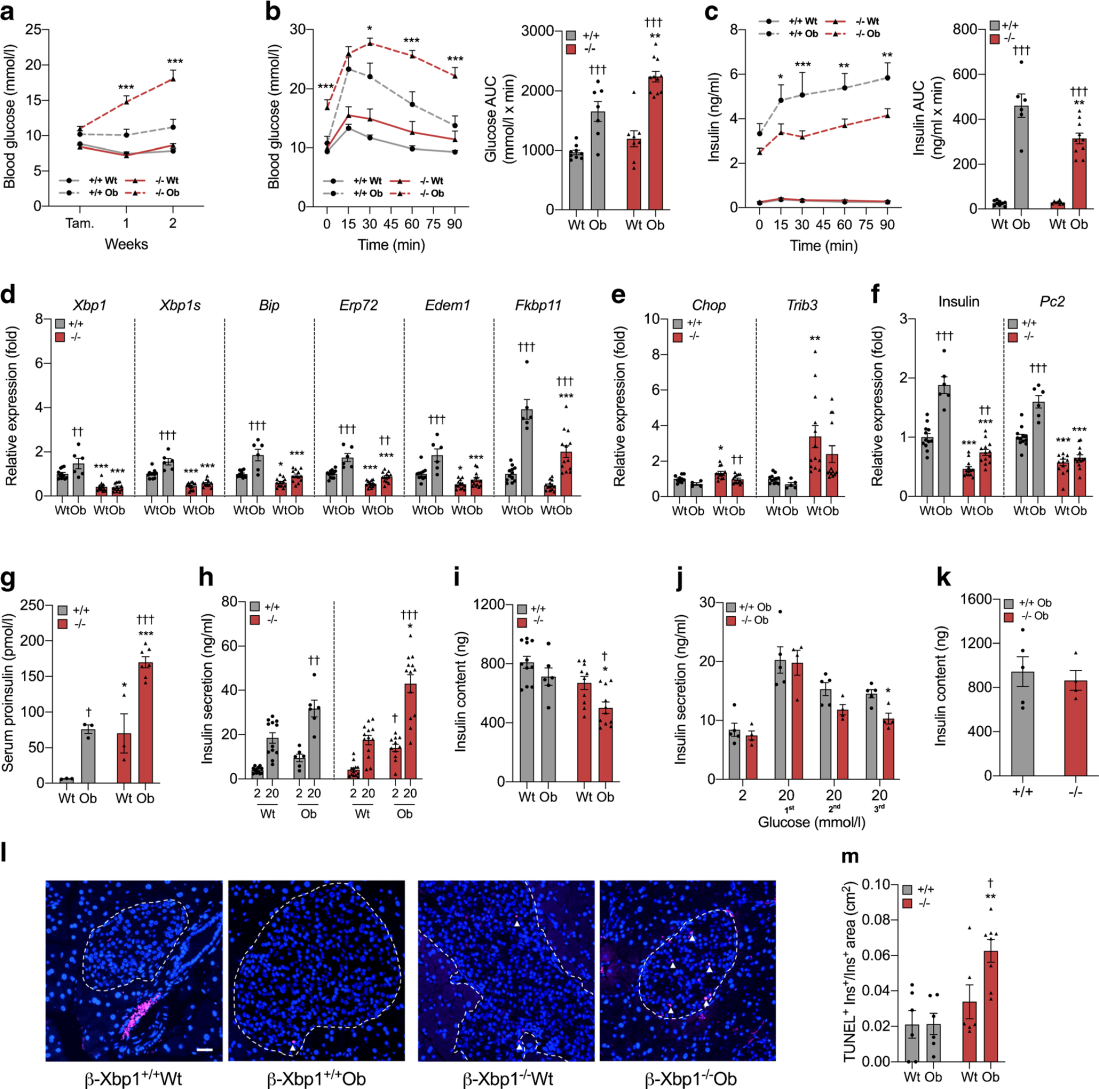
Beta cell-specific *Xbp1* deletion in obese o*b/ob* mice leads to diabetes due to failure of beta cell compensation. (**a**) Fed blood glucose levels. (**b**) Blood glucose levels and AUC for glucose during OGTT. (**c**) Serum insulin levels and AUC for insulin during OGTT. (**d**) mRNA expression of adaptive UPR genes in islets expressed as fold change of the levels in β-Xbp1^+/+^Wt mice. (**e**) mRNA expression of pro-apoptosis UPR genes in islets expressed as fold change of the levels in β-Xbp1^+/+^Wt mice. (**f**) mRNA expression of insulin and *Pc2* in islets expressed as fold change of the levels in β-Xbp1^+/+^Wt mice. (**g**) Serum proinsulin levels. (**h**) Insulin secretion from isolated islets treated with low (2 mmol/l) or high (20 mmol/l) stimulatory level of glucose for 1 h. (**i**) Insulin content in islets. (**j**) Insulin secretion from isolated islets of β-Xbp1^+/+^Ob and β-Xbp1^−/−^Ob mice treated with repeated low (2 mmol/l) or high (20 mmol/l) stimulatory level of glucose for three cycles. (**k**) Insulin content in islets following three cycles of glucose treatment. (**l**) Representative images of TUNEL immunostaining in pancreas sections. (**m**) Beta cell apoptosis rate (quantification of immunostaining for TUNEL and insulin). All data are represented as means ± SEM. (**a**–**i**) *n* = 3–12, β-Xbp1^+/+^Wt; *n* = 3–14, β-Xbp1^−/−^Wt; *n* = 3–7, β-Xbp1^+/+^Ob; *n* = 7–13, β-Xbp1^−/−^Ob. (**j**, **k**) *n* = 5, β-Xbp1^+/+^Ob; *n* = 4, β-Xbp1^−/−^Ob. (**l**, **m**) *n* = 6, β-Xbp1^+/+^Wt; *n* = 6, β-Xbp1^−/−^Wt; *n* = 6, β-Xbp1^+/+^Ob; *n* = 8, β-Xbp1^−/−^Ob. ANOVA: *p<0.05, **p<0.01, ***p<0.001 *Xbp1* genotype effect; ^†^*p*<0.05, ^††^*p*<0.01, ^†††^*p*<0.001 Ob genotype effect. Tam., tamoxifen

To test whether XBP1 expression makes a necessary contribution to in vivo beta cell compensation and diabetes resistance in obese mice, we crossed *Xbp1*^flox/flox^ *Pdx1*-Cre^ER^ and *ob*/+ mice to generate *Xbp1*^flox/flox^ *Pdx1*-Cre^ER^ *ob/ob* (β-Xbp1^−/−^Ob) and *Xbp1*^flox/flox^ *Pdx1*-Cre^ER^ (β-Xbp1^−/−^Wt) mice and control *Xbp1*^+/+^ *Pdx1*-Cre^ER^ *ob/ob* (β-Xbp1^+/+^Ob) and *Xbp1*^+/+^ *Pdx1*-Cre^ER^ (β-Xbp1^+/+^Wt) mice. *Xbp1* deletion was induced in 8–10-week-old mice by tamoxifen administration. Body weight was increased in the groups of *ob/ob* mice (β-Xbp1^+/+^Ob and β-Xbp1^−/−^Ob) compared with respective lean controls (β-Xbp1^+/+^Wt and β-Xbp1^−/−^Wt), but was not affected by *Xbp1* deletion (ESM Fig. 6). Fed blood glucose levels in obese β-Xbp1^−/−^Ob mice were significantly increased 1 and 2 weeks after *Xbp1* deletion compared with all other groups (Fig. 3a), demonstrating that *Xbp1* deletion leads to diabetes under conditions of obesity. Obese β-Xbp1^+/+^Ob mice compared with lean β-Xbp1^+/+^Wt mice, as expected, were glucose intolerant, as shown by the OGTT blood glucose levels and glucose AUC (Fig. 3b). Elevated fasting glucose and more severe glucose intolerance were observed in β-Xbp1^−/−^Ob mice (Fig. 3b). Insulin levels and the insulin AUC during the OGTT were significantly increased in obese β-Xbp1^+/+^Ob mice compared with lean β-Xbp1^+/+^Wt mice (Fig. 3c), characteristic of the beta cell compensatory response to obesity. Insulin excursions, however, were significantly reduced in β-Xbp1^−/−^Ob mice compared with β-Xbp1^+/+^Ob mice (Fig. 3c). *Xbp1* deletion may therefore result in impaired compensatory insulin secretion under conditions of obesity, causing diabetes.

In islets isolated from obese β-Xbp1^+/+^Ob mice, the mRNA levels of *Xbp1* and the adaptive UPR genes were increased compared with lean β-Xbp1^+/+^Wt mice (Fig. 3d), thus confirming that adaptive UPR activation is associated with beta cell compensation in obese mice. As expected, the expression levels of *Xbp1* and spliced *Xbp1* were significantly reduced in islets of β-Xbp1^−/−^Wt and β-Xbp1^−/−^Ob mice compared with respective control mice (Fig. 3d). This was associated with downregulation of *Bip*, *Erp72*, *Edem1* and *Fkbp11* (Fig. 3d), thus demonstrating that *Xbp1* deletion reduces expression of adaptive UPR genes in islets of lean mice and results in their impaired activation in islets of obese mice. On the other hand, the expression levels of the deleterious UPR genes, *Chop* and *Trib3*, were increased in islets of β-Xbp1^−/−^Wt compared with β-Xbp1^+/+^Wt mice, with suggestion of partial attenuation of this effect in β-Xbp1^−/−^Ob mice (Fig. 3e). Thus, unexpectedly, the inhibition of XBP1-mediated adaptive UPR in islets of obese β-Xbp1^−/−^Ob mice was not associated with a pro-apoptotic UPR activation (Fig. 3e).

The expression of insulin and *Pc2* was significantly induced in the islets of obese β-Xbp1^+/+^Ob mice compared with lean β-Xbp1^+/+^Wt mice (Fig. 3f), providing evidence of an improved beta cell capacity to meet greater insulin demand in obesity. However, insulin and *Pc2* mRNA levels were significantly reduced following *Xbp1* deletion in both lean β-Xbp1^−/−^Wt and obese β-Xbp1^−/−^Ob mice (Fig. 3f). The highest levels of serum proinsulin among the groups were observed in obese β-Xbp1^−/−^Ob mice (Fig. 3g), thus confirming the significance of diminished beta cell capacity for proinsulin processing in the setting of high insulin demand.

The expected compensatory increase in glucose-stimulated insulin secretion in obesity was observed in islets isolated from β-Xbp1^+/+^Ob mice (Fig. 3h). Surprisingly, this response was enhanced in islets from β-Xbp1^−/−^Ob mice (Fig. 3h), implying that under static ex vivo conditions secretory mechanisms may be preserved with *Xbp1* deletion. We hypothesised that challenge with a single glucose exposure might not be sufficient to reveal limitations on secretion. We therefore subjected islets from β-Xbp1^+/+^Ob and β-Xbp1^−/−^Ob mice to repeated high-glucose exposure to mimic the metabolic challenge caused by hyperphagia in obese mice [34]. After three cycles of high-glucose stimulation, β-Xbp1^+/+^Ob islets displayed only a slight non-significant decline in insulin secretion, whereas insulin secretion in β-Xbp1^−/−^Ob islets was significantly reduced by ~50% (Fig. 3j), with unchanged insulin content (Fig. 3k). Similar results were obtained using β-Xbp1^−/−^Wt islets, although with reduced insulin content (ESM Fig. 7). Thus, islets with *Xbp1* deletion were susceptible to insulin secretory dysfunction after repeated metabolic challenge.

### *Xbp1* deletion in beta cells of o*b/ob* mice leads to increased beta cell apoptosis

The incidence of TUNEL-positive beta islets in obese β-Xbp1^+/+^Ob mice was not different from control β-Xbp1^+/+^Wt mice, suggesting that beta cell apoptosis was not altered in diabetes-resistant *ob/ob* mice (Fig. 3l,m). In contrast, the incidence of TUNEL-positive cells, although unchanged in β-Xbp1^−/−^Wt mice, was significantly increased by threefold in β-Xbp1^−/−^Ob mice (Fig. 3l,m), suggesting that the absence of XBP1 increases the rate of beta cell apoptosis under conditions of obesity. Notably, the findings reveal that the variations in beta cell apoptosis did not correlate with the changes in pro-apoptotic UPR, which raises the important question: what is the mechanism(s) of increased beta cell apoptosis following XBP1 loss under conditions of metabolic challenge?

### *Xbp1* deletion increases beta cell apoptosis by attenuating the antioxidant response under metabolic stress conditions

**Fig. 4:**
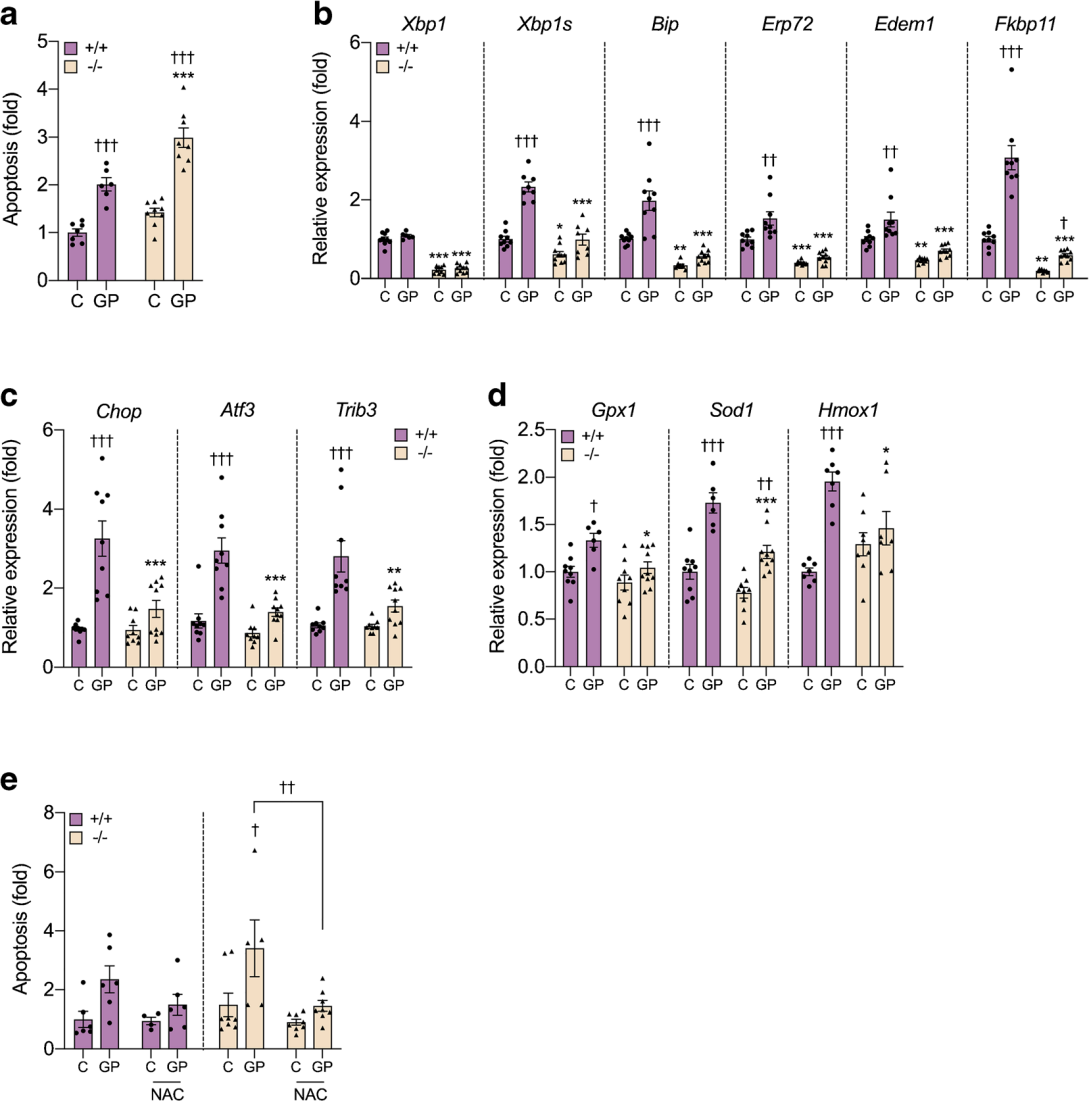
*Xbp1* deletion in beta cells potentiates glucolipoapoptosis by attenuating the antioxidant response. (**a**) Apoptosis rate in islets determined by cell death detection ELISA. (**b**) mRNA expression of adaptive UPR genes in islets expressed as fold change of the levels in β-Xbp1^+/+^ islets. (**c**) mRNA expression of pro-apoptosis UPR genes in islets expressed as fold change of the levels in β-Xbp1^+/+^ islets. (**d**) mRNA expression of antioxidant enzymes in islets expressed as fold change of the levels in β-Xbp1^+/+^ islets. (**e**) Apoptosis rate in islets co-treated in the absence or presence of the antioxidant. Islets were treated in the absence (control, C) or presence of high GP for 72 h. All data are represented as means±SEM. *n* = 6–9, β-Xbp1^+/+^; *n* = 8–9, β-Xbp1^−/−^. ANOVA: *p<0.05, **p<0.01, ***p<0.001 genotype effect; ^†^*p*<0.05, ^††^*p*<0.01, ^†††^*p*<0.001 treatment effect

To address this question, we established a model of ex vivo glucolipotoxicity using β-Xbp1^+/+^ and β-Xbp1^−/−^ islets treated with high glucose (25 mmol/l) + saturated fatty acid palmitate (0.4 mmol/l coupled to 0.92% BSA) for 72 h (termed GP). Notably, the twofold increase in apoptosis observed in β-Xbp1^+/+^ islets after GP exposure was significantly potentiated in β-Xbp1^−/−^ islets (Fig. 4a). The expression levels of spliced *Xbp1* and the adaptive UPR genes, *Bip*, *Erp72*, *Edem1* and *Fkbp11*, were increased after GP exposure of β-Xbp1^+/+^ islets (Fig. 4b). However, in β-Xbp1^−/−^ islets, the expression levels of *Xbp1* and spliced *Xbp1* were significantly reduced along with the adaptive UPR genes in islets treated under control conditions, and their activation by GP treatment was markedly attenuated (Fig. 4b). The mRNA levels of the deleterious UPR genes, *Chop*, *Atf3* and *Trib3*, were strongly increased by GP treatment in control β-Xbp1^+/+^ islets, whereas a comparatively blunted response was observed in β-Xbp1^−/−^ islets (Fig. 4c). These results with ex vivo glucolipoapoptosis closely resemble the in vivo findings, suggesting that the potentiation of beta cell apoptosis following XBP1 loss in metabolic stress conditions occurs in concert with lowered pro-apoptotic UPR.

Oxidative stress has been implicated in beta cell apoptosis in type 2 diabetes [35] and has been linked with UPR deregulation [36]. We therefore examined whether changes in redox status could contribute to the potentiation of glucolipoapoptosis in β-Xbp1^−/−^ islets. GP treatment significantly increased the expression of several antioxidant enzymes, heme-oxygenase-1 (*Hmox1*), superoxide dismutase-1 (*Sod1*) and glutathione peroxidase (*Gpx1*), in β-Xbp1^+/+^ islets (Fig. 4d), consistent with activation of a robust adaptive response to oxidative stress. In contrast, the antioxidant mRNA levels were significantly reduced in GP-treated β-Xbp1^−/−^ islets compared with β-Xbp1^+/+^ islets (Fig. 4d), suggesting that *Xbp1* deletion inhibited the antioxidant response. We next co-treated islets with NAC, a commonly used antioxidant that acts as a reduced glutathione precursor [37]. NAC co-treatment completely rescued β-Xbp1^−/−^ islets from the potentiation of glucolipoapoptosis (Fig. 4e). These findings suggest that XBP1 protects against beta cell apoptosis during metabolic stress by promoting an antioxidant response.

## Discussion

Our data demonstrate that XBP1 plays an essential role in the maintenance of beta cell identity, in the repression of beta-to-alpha cell reprogramming and in preserving normoglycaemia in the face of insulin resistance by facilitating beta cell adaptation. Loss of XBP1 resulted in beta cell dedifferentiation, as evidenced by: (1) downregulation of beta cell-enriched genes; (2) concomitant upregulation of beta cell forbidden genes; and (3) appearance of alpha cell features in lineage-labelled beta cells. The maladaptation of XBP1-deficient beta cells under metabolic stress conditions was associated with deterioration of beta cell dysfunction and increased beta cell apoptosis due to impaired handling of oxidative stress. Thus, our studies suggest that the decline in spliced XBP1 expression found in type 2 diabetes [29] contributes to the failure of beta cells to secrete sufficient amounts of insulin to meet metabolic demand.

Primary defects induced by XBP1 deficiency in the absence of diabetes and, thus, independently of glucotoxicity, include beta cell dedifferentiation, beta-to-alpha cell conversion and impaired proinsulin processing. These underlying defects rendered beta cells incapable of adapting to metabolic challenge, triggering hyperglycaemia and further decline of beta cell capacity. Glucotoxicity [38], inflammatory stress [39] and oxidative stress [35] may play important roles in beta cell deterioration and development of secondary abnormalities, which include islet inflammation, cellular senescence and increased beta cell apoptosis.

Previous studies showed that embryonic deletion of *Xbp1* in beta cells led to reduced islet area in association with impaired beta cell proliferation [21]. In stark contrast, the deletion of *Xbp1* in adult beta cells resulted in increased proliferation, while beta cell loss under metabolic stress conditions was due to increased apoptosis. Moreover, previous studies in MIN6 cells led to the conclusion that XBP1 deficiency resulted in feedback inositol-requiring enzyme 1 α (IRE1α) hyperactivation [21]. However, we found unchanged IRE1α phosphorylation in islets isolated from *Xbp1* knockout mice fed a chow diet compared with control mice (ESM Fig. 8). Furthermore, the expression of *Atf4*, a transcription factor induced downstream of PERK, was unchanged (ESM Fig. 9). Instead, our data demonstrate that beta cell dedifferentiation induced by *Xbp1* deletion was associated with altered expression of multiple transcriptional regulators of beta cell identity (*Pdx1*, *Beta2*, *Nkx6.1* and *Foxo1*) [8, 40–46], and increased expression of *Myc*, a cell cycle regulator that promotes beta cell replication, but concomitantly diverts beta cells towards an immature phenotype [32, 47, 48]. Chronic hyperglycaemia, islet cell aggregation and several specific genetic manipulations can lead to conversion of beta cells to alpha cells [8, 10, 11, 46, 49–57]. To our knowledge, this is the first study showing that a UPR factor can drive the process of beta-to-alpha cell transdifferentiation.

IRE1α has been implicated in the regulation of insulin biosynthesis [27, 58–60]. Compared with our model of *Xbp1* deletion, IRE1α deletion in beta cells of adult mice caused a significantly greater diabetic phenotype in association with increased production of reactive oxygen species, but without reducing expression of beta cell-specific mRNAs [60]. Moreover, a recent study showed that IRE1α deletion in the non-obese diabetic (NOD) mouse model of type 1 diabetes induced transient hyperglycaemia and beta cell dedifferentiation, which protected against immune-mediated destruction [61]. Our study highlights a requirement of XBP1 in activation of antioxidant genes that promote beta cell survival under metabolic stress conditions.

In summary, we present the first evidence that XBP1 maintains mature beta cell identity, represses beta-to-alpha cell transdifferentiation and is required for beta cell protection against diabetes in insulin resistance states. From a therapeutic perspective, targeting XBP1 might help to reverse the process of beta cell dedifferentiation and restore functional beta cell mass in type 2 diabetes.

## Supplementary information

ESM (PDF 1077 kb)

## Data Availability

All data generated and analysed during this study are included in this published article (and its supplementary material files).

## Abbreviations

ER: Endoplasmic reticulum
GP: Glucose + palmitate
IRE1: Inositol-requiring enzyme 1
IRE1α: Inositol-requiring enzyme 1 α
NAC: *N*-acetyl-l-cysteine
PERK: PKR-like ER kinase
TRAP: Translating ribosome affinity purification
UPR: Unfolded protein response
XBP1: X-box binding protein 1
