# Supplementary material for: XBP1 maintains beta cell identity, represses beta-to-alpha cell transdifferentiation and protects against diabetic beta cell failure during metabolic stress in mice
Source: Diabetologia. 2022 Mar 22;65(6):984–96. doi: 10.1007/s00125-022-05669-7 (PMC9076738; doi:10.1007/s00125-022-05669-7)
Supplement: Supplementary file 1 — (PDF 1077 kb) [file 125_2022_5669_MOESM1_ESM.pdf]

ELECTRONIC SUPPLEMENTARY MATERIAL (ESM)

XBP1 maintains beta cell identity, represses beta-to-alpha cell transdifferentiation and protects against diabetic beta cell failure during metabolic stress in mice

ESM Methods

Mouse models and maintenance

All research and animal care procedures were approved by the Garvan Institute/St.Vincent's Hospital Animal Experimentation Ethics Committee, following guidelines issued by the National Health and Medical Research Council of Australia. Mice were group-housed (2-5 mice per cage) under conditions of controlled temperature (22 °C) and illumination (12 h light cycle, lights on at 07:00 h). Mice had free access to water and were fed a standard chow diet (8 % calories from fat, 21 % calories from protein, 71 % calories from carbohydrate, 10.88 kJ/g; Gordon's Specialty Stock Feeds, Yanderra, NSW, Australia).

To enable tamoxifen-inducible beta cell specific deletion of XBP1, we crossed Pdx1-Cre^{ER} mice [Tg(Pdx1-cre/Esr1*)#Dam/J; The Jackson Laboratory, Bar Harbour, ME, USA] with Xbp1^{flox} mice kindly provided by Laurie Glimcher, Dana-Farber Cancer Institute, Harvard University, Boston, MA, USA [1]. Experimental mice received i.p. tamoxifen (Sigma-Aldrich, St Louis, MO, USA) injections (3 x 75 mg/kg body weight) at 8-10 weeks of age to generate Xbp1$^{flox/flox}$ Pdx1-Cre^{ER} (denoted β-Xbp1$^{-/-}$) and littermate control Xbp1$^{+/+}$ Pdx1-Cre^{ER} (denoted β-Xbp1$^{+/+}$) mice. Littermates were assigned randomly to either the chow diet (8 % calories from fat, 21 % calories from protein, 71 % calories from carbohydrate, 10.88 kJ/g; Gordon's Specialty Stock Feeds, Yanderra, NSW, Australia) or a lard-based high-fat diet prepared in-house (19.67 kJ/g; 45% fat, 20% protein and 35% carbohydrate [16% sucrose]; based on Research Diets D12451, New Brunswick, NJ, USA) for up to 5 weeks.

To enable tamoxifen-inducible beta cell-specific deletion of XBP1 in obese mice, we first crossed Xbp1$^{flox/flox}$ Pdx1-Cre^{ER} mice with *ob*/+ (B6.Cg-*Lep*^{ob}/J; The Jackson Laboratory) mice. Then Xbp1$^{flox/+}$ Pdx1-Cre^{ER} *ob*/+ mice were mated to generate littermate Xbp1$^{flox/flox}$ Pdx1-Cre^{ER} ob/ob (β-Xbp1$^{-/-}$Ob) and Xbp1$^{flox/flox}$ Pdx1-Cre^{ER} wildtype/wildtype (β-Xbp1$^{-/-}$Wt) mice and control Xbp1$^{+/+}$ Pdx1-Cre^{ER} ob/ob (β-Xbp1$^{+/+}$Ob) and Xbp1$^{+/+}$ Pdx1-Cre^{ER} wildtype/wildtype (β-Xbp1$^{+/+}$Wt) mice. Experimental mice received i.p. tamoxifen injections (3 x 75 mg/kg body weight) at 8-10 weeks of age. Littermates were fed a chow diet (8 % calories from fat, 21 % calories from protein, 71 % calories from carbohydrate, 10.88 kJ/g; Gordon's Specialty Stock Feeds, Yanderra, NSW, Australia).

A model for cell-type-specific lineage tracing and transcript profiling was developed by crossing Xbp1$^{flox/flox}$ Pdx1-Cre^{ER} mice with Gt/Rosa26^{GFP} [Gt(ROSA)26Sor$^{tm9(EGFP/Rpl10a)Amc}$; The Jackson Laboratory] mice. Mice received i.p. tamoxifen injections (3 x 75 mg/kg body weight) at 8-10 weeks of age to generate Xbp1$^{flox/flox}$ Pdx1-Cre^{ER} Gt/Rosa26^{GFP} (β-Xbp1$^{-/-}$Gt) and control Xbp1$^{+/+}$ Pdx1-Cre^{ER} Gt/Rosa26^{GFP} (β-Xbp1$^{+/+}$Gt) mice. Mice were fed a chow diet (8 % calories from fat, 21 % calories from protein, 71 % calories from carbohydrate, 10.88 kJ/g; Gordon's Specialty Stock Feeds, Yanderra, NSW, Australia) for 5 weeks.

Powdered tamoxifen was dissolved in filtered sunflower oil by gentle rotation for 3 h at 37 °C avoiding light. Tamoxifen solution (20 mg/ml) was prepared fresh prior to use. Mice received 3 x 75 mg tamoxifen/kg body weight over 5 days via i.p. injection.

Glucose tolerance test

Mice were fasted for 6 h, weighed and basal blood samples taken from the tail tip. They were orally administered by gavage with a 50% D-glucose solution (1.5 or 3 g/kg body weight, Phebra, Lane Cove, NSW, Australia) and blood samples were taken at 15, 30, 60 and 90 min. Blood glucose was measured using an AccuCheck Performa glucometer (Roche, Castle Hill, NSW, Australia). 5 µl of blood was added to 95 µl of sample diluent and insulin was measured by ELISA (Crystal Chem, Downers Grove, IL, USA). Proinsulin was measured in mouse plasma using the Proinsulin Rat/Mouse ELISA kit (Mercodia, Uppsala, Sweden).

Islet isolation

Pancreas was perfused via the common bile duct with cold Liberase solution (Krebs-Ringer HEPES buffer, KRB-HEPES: 125 mmol/l NaCl, 4.8 mmol/l KCl, 1 mmol/l $CaCl_2$, 1.2 mmol/l $KH_2(PO)_4$, 1.18 mmol/l $MgSO_4$, 5 mmol/l $NaHCO_3$ and 25 mmol/l HEPES, supplemented with 2.8 mmol/l glucose and 0.25 mg/ml Liberase (Roche). The pancreas was removed and incubated at 37°C for 16 min. The digestion was stopped by addition of KRB-HEPES containing 10 % newborn calf serum followed by mechanical disruption of the pancreas and washing of the islets. Islets were further separated using a Ficoll-Paque PLUS (GE Healthcare, Uppsala, Sweden) density gradient with centrifugation followed by handpicking under a stereomicroscope.

Glucose-stimulated insulin secretion *ex vivo*

Experiments were performed in batches of 5 islets in triplicate. Islets were pre-incubated in 100 µl of KRB-HEPES supplemented with 0.1 % BSA and 2 mmol/l glucose for 30 min at 37°C in a 96-well V-bottom microplate. Then they were incubated in 100 µl of fresh KRB-HEPES supplemented with 0.1 % BSA containing either 2 or 20 mmol/l glucose for 1 h at 37°C. An aliquot of the buffer was taken, and secreted insulin determined using the Insulin Ultra-Sensitive Assay (Cisbio, Codolet, France). The islets were lysed for measurement of insulin and DNA content.

Glucolipotoxicity and antioxidant treatment *ex vivo*

Islets isolated from littermate $Xbp1^{+/+}$ $Pdx1\text{-}Cre^{ER}$ and $Xbp1^{flox/flox}$ $Pdx1\text{-}Cre^{ER}$ mice were cultured in islet media (RPMI 1640 medium, supplemented with 0.2 mmol/l glutamine, 10 % heat-inactivated fetal bovine serum (FBS), 100 U/ml penicillin and 100 µg/ml streptomycin) at 37°C overnight. To enable beta cell-specific deletion of XBP1, islets were treated with 100 nmol/l 4-hydroxy tamoxifen (Sigma-Aldrich) to generate $\beta\text{-}Xbp1^{+/+}$ and $\beta\text{-}Xbp1^{-/-}$ islets. Next, islets were treated in islet media with either 0.92 % BSA (control) or 25 mmol/l glucose and 0.4 mmol/l palmitate coupled to 0.92 % BSA (termed glucose + palmitate, GP) for 72 h at 37°C. For antioxidant treatment experiments, islets were co-treated with 2.5 mmol/l *N*-acetyl-L-cysteine (Sigma-Aldrich). Cell death was determined in islets using the Cell Death Detection $ELISA^{PLUS}$ Kit (Roche Diagnostics). The assay measures cytoplasmic histone-bound DNA fragments (mono- and oligonucleosomes) after apoptotic cell death.

Histology and immunohistochemistry

Pancreases were weighed and fixed in 10 % neutral-buffered formalin solution overnight at 4°C, and then stored in 70 % ethanol at 4°C. The pancreata were embedded in paraffin and sections were cut at 4-5 µm for histology and immunohistochemistry. Sections were deparaffinised and rehydrated using Leica Autostainer (Leica ST5010, Leica Microsystems, Wetzlar, Germany). Slides were baked for 4 min at 60°C and dewaxed in Xylene twice for 3 min. Then, slides were washed in 100 % ethanol for 3 min and rehydrated by washing in 95 %, 70 % and 50 % ethanol for 3 min each. Slides were then rinsed in distilled water. Heat-induced antigen retrieval was performed using Targeted retrieval solution (S1699) and heating the slides to 125°C for 1 min and 95°C for 10 sec in a pressure cooker. Alternatively, enzymatic antigen retrieval with proteinase K at 37°C for 30 min was used for glucagon staining. Slides were then washed in PBS and TBST (0.1 % Tween-20) for 5 min and permeabilised in TBS + 1 % Triton X-100 for 5 min. They were then rinsed in TBST 3 times for 5 min to remove the triton solution. The slides were then blocked with PBS containing 2 % BSA and 5 % goat serum for 30 min at room temperature and then incubated with respective primary antibody overnight at 4°C (ESM Table 1). The primary antibody was removed and slides washed with TBST 3 times, followed by incubation with the secondary antibody for 1 h at room temperature avoiding light (ESM Table 1). The secondary antibody was washed with TBST 3 times and the slides mounted with a DAPI mounting media (ProLong Gold, Life Technologies, Carlsbad, CA, USA), cover slipped and allowed to dry. For *in situ* apoptosis detection, formalin-fixed, paraffin-embedded pancreatic sections were stained using mouse anti-insulin antibody and the Click-iT™ Plus TUNEL Assay (ThermoFisher Scientific, Scoresby, VIC, Australia) according to manufacturer's instructions. Sections were mounted with DAPI mounting media (ProLong Gold), cover slipped and allowed to dry. Slides were imaged using a Leica DM5500 fluorescent microscope or Leica DM6000 Power Mosaic microscope (Leica Microsystems).

Immuno-morphometry

Immunostaining was quantified using semi-automated scripts and plugin commands written for Image J/FIJI (ImageJ Developers and FIJI Contributors). Immunofluorescent images of insulin and glucagon staining were used to outline an insulin-defined and glucagon-defined region of interest (ROI), respectively. ROIs were used to calculate beta cell and alpha cell area (μm^2) for each section, and beta cell and alpha cell mass calculated using the area and total pancreas mass. Beta cell proliferation and apoptosis were assessed by overlaying the insulin-defined ROI with images of DAPI, Ki-67 and TUNEL stained sections. Three pancreas sections separated by at least 100 µm were used for quantification.

Electron microscopy

Freshly isolated islets were fixed in 2 % glutaraldehyde in 0.1 M cacodylate buffer, pH 7.4, overnight at 4°C. After consecutive washes in 0.1 M cacodylate buffer, the islet pellets were postfixed in 4 % osmium in the cacodylate buffer for 1 h at room temperature. Two washes in distilled water were followed by a block staining with 2 % aqueous uranyl acetate. The islets were then dehydrated through a series of ethanol and embedded in TAAB epoxy resin. Ultrathin sections (90 nm) were cut, mounted on 150-mesh copper/palladium grids and stained with Reynolds lead citrate. 10-12 representative digital electron micrographs of individual beta cells were captured from 5-6 different islets under transmission electron microscope (JEOL 1011; JEOL Ltd, Tokyo, Japan) using a digital camera (MegaView G2;

Olympus, Münster, Germany). Images were analyzed with iTEM software v.5.2 (Olympus). Mature and immature insulin granules (IG) were identified based on core density and presence of characteristic halo.

Immunoblotting

Islets were mechanically homogenized in lysis buffer (50 mmol/l HEPES, 150 mmol/l NaCl, 10 % glycerol, 1 % Triton X-100, 1.5 mmol/l $MgCl_2$, 1 mmol/l EGTA, 10 mmol/l $Na_4P_2O_7$ and 100 mmol/l NaF) supplemented with Complete protease inhibitor (Roche) and 200 µmol/l $Na_3VO_4 \cdot 2H_2O$. Lysates were then centrifuged at 13,200 RPM for 10 min at 4°C. Supernatants were assayed for protein concentration using the BCA Protein Assay Kit (ThermoFisher Scientific) according to the manufacturer's instructions. Equal quantities of protein lysates (20-50 µg) were mixed with NuPAGE LDS 4x Sample Buffer and 10x Sample Reducing Agent (ThermoFisher Scientific). Samples were denatured at 70°C for 10 min and then they were resolved by pre-cast NuPAGE or Criterion SDS-PAGE gels for 60-80 min at 150 V in MOPS running buffer (50 mmol/l MOPS, 50 mmol/l Tris base, 0.1 % SDS, 1 mmol/l EDTA) supplemented with NuPAGE Antioxidant (ThermoFisher Scientific). Proteins in gels were electrotransferred to Immobilon P PVDF membranes (Millipore, Billerica, MA, USA) in Tris-glycine buffer (25 mmol/l Tris, 192 mmol/l glycine, 10% methanol and 0.025 % SDS) using a Trans-Blot Cell (Bio-Rad) at 400 mA or Mini Trans-Blot cell (Bio-Rad, Hercules, CA, USA) at 90 V for 2 h. Membranes were blocked in a TTBS solution (10 mM Tris, 150 mmol/l NaCl, 0.05 % Tween-20) containing 5% skim milk for 2 h. Membranes were washed and then incubated overnight at 4°C with primary antibodies (ESM Table 1) in TTBS containing 5 % BSA or skim milk. After washing 4 times in TTBS over 1.5 h, membranes were incubated in secondary antibodies (sheep anti-mouse or donkey anti-rabbit, 1:5000 dilution) conjugated to horseradish peroxidise (HRP) at room temperature for 1 h. Membranes were exposed to Western Lightning Plus-ECL substrate (Perkin Elmer) and images captured on a Super RX X-ray film (FujiFilm). Films were scanned with an Epson Perfection V800 Photo scanner and bands quantified by densitometry using ImageJ software (ImageJ Developers).

RNA analysis

Total RNA from islets was extracted using RNeasy Mini Kit according to manufacturer's instructions (Qiagen, Doncaster, VIC, Australia). RNA concentration was determined using a Nanodrop spectrophotometer. RNA (200 ng) was reverse transcribed to cDNA using the QuantiTect Reverse Transcription Kit (Qiagen) according to manufacturer's instructions. Real-time PCR was performed in a 384-well plate on the 7900 HT Real Time PCR System (Applied Biosystems, Foster City, CA, USA) using standard reaction cycle conditions. The 10 µl reaction volume consisting of cDNA, 0.6 µmol/l oligonucleotide primers (ESM Table 2) and PowerSYBR Green master mix was prepared using automated pipetting on the epMotion 5070 (Eppendorf, Hamburg, Germany). The value obtained for each specific gene product was normalised to a housekeeping gene (cyclophilin A) and expressed as a fold-change of the value in control extracts.

Translating ribosome affinity purification

Translating ribosome affinity purification (TRAP) was performed based on the previously published protocol with some modifications [2, 3]. Islets isolated from β-$Xbp1^{+/+}$Gt and β-$Xbp1^{-/-}$Gt mice were homogenized in 500 µl lysis buffer (20 mM HEPES-KOH [pH 7.4], 5 mmol/l $MgCl_2$, 150 mmol/l KCl) supplemented with 0.5 mmol/l DTT, fresh protease inhibitor (1

tablet/ml; Roche Mini Complete, EDTA-Free), 100 µg/ml cycloheximide and 40 U/ml Rnasin (Promega, Madison, WI, USA). After lysis, homogenates were centrifuged for 10 min at 2000 x G at 4°C to remove pellet nuclei and cell debris. 50 µl of 10 % NP-40 working solution (Biochemica) was added to the supernatant and mixed by gentle inversion. Next, 50 µl of DHPC (300 mmol/l) was added to the supernatant and mixed by gentle inversion and incubated on ice for 5 min. The lysate was centrifuged for 10 min at 13,000 x G. 20% (100 µl) of the supernatant was kept as input. For preparation of the GFP antibody-Dynabeads solution, 50 µl of protein G Dynabeads (Invitrogen, Carlsbad, CA, USA) was rinsed three times with 1 ml of 0.15 mol/l KCl buffer (20 mmol/l HEPES-KOH [pH 7.4], 5 mmol/l $MgCl_2$, 150 mmol/l KCl, 1 % NP-40, 0.5 mmol/l DTT, 100 µg/ml cycloheximide) at room temperature. Then 5 µl of anti-GFP antibody (2 µg/µl; Invitrogen) was added to the beads and incubated with 275 µl of 0.15 mol/l KCl buffer for 1 h at room temperature with slow end-to-end rotation. Next, the antibody-bound beads were collected using a magnetic rack and the supernatant discarded. The collected beads were washed three times with 0.15 mol/l KCl buffer. The antibody-bead complex was resuspended in 200 µl of 0.15 mol/l KCl buffer before use. The beads were then mixed with the cell-lysate supernatant, and the mixture was incubated at 4°C with slow end-to-end rotation overnight. The complex-bound Dynabeads were collected with a magnetic rack, washed 3 times with 0.35 mol/l KCl buffer (20 mmol/l HEPES-KOH [pH 7.4], 5 mmol/l $MgCl_2$, 350 mmol/l KCl, 1 % NP-40, 0.5 mmol/l DTT, 100 µg/ml cycloheximide) at 4°C, and immediately resuspended in 350 µl of RLT buffer supplemented with 10 % 2-mercaptoethanol and incubated for 5 min at room temperature. The beads were removed using the magnetic rack and the RLT-containing RNA was precipitated with equal parts of 70% ethanol and then purified using the RNeasy Micro Kit (Qiagen) according to manufacturer's instructions. RNA concentration was determined using a Nanodrop spectrophotometer. 40 ng of RNA was used to synthesize cDNA using the QuantiTect Reverse Transcription Kit (Qiagen) according to manufacturer's instructions for subsequent RT-PCR analysis.

Statistical analysis

All data are represented as means ± SEM. Unpaired two-tailed *t*-test was used to compare differences between two groups. Differences between more than two groups were calculated using two-way ANOVA with Tukey's post-hoc test.

References

[1] Lee AH, Heidtman K, Hotamisligil GS, Glimcher LH (2011) Dual and opposing roles of the unfolded protein response regulated by IRE1alpha and XBP1 in proinsulin processing and insulin secretion. Proc Natl Acad Sci U S A 108(21): 8885-8890. 10.1073/pnas.1105564108

[2] Heiman M, Kulicke R, Fenster RJ, Greengard P, Heintz N (2014) Cell type-specific mRNA purification by translating ribosome affinity purification (TRAP). Nature protocols 9(6): 1282-1291. 10.1038/nprot.2014.085

[3] Ip CK, Zhang L, Farzi A, et al. (2019) Amygdala NPY Circuits Promote the Development of Accelerated Obesity under Chronic Stress Conditions. Cell Metab 30(1): 111-128 e116. 10.1016/j.cmet.2019.04.001

ESM Table 1. Antibodies

Antibody	Source	Identifier
anti-GFP polyclonal (1:500)	Invitrogen	Cat# A-11122
anti-Glucagon monoclonal (1:50)	Abcam	Cat# AB18461
anti-Glucagon monoclonal (1:500)	Sigma	Cat# G2654
anti-Insulin polyclonal (1:50)	Abcam	Cat# AB7842
anti-Insulin monoclonal (1:500)	Sigma	Cat# I2018
anti-IRE1 polyclonal (1:5000)	Abcam	Cat# AB37073
anti-IRE1α (phospho S724) polyclonal (1:5000)	Abcam	Cat# AB48187
anti-Ki-67 monoclonal (1:250)	Abcam	Cat# AB16667
anti-XBP1 polyclonal (1:5000)	Santa Cruz	Cat# sc-7160
anti-14-3-3 monoclonal (H-8) (1:5000)	Santa Cruz	Cat# sc-1657
Alexa 488 anti-mouse secondary (1:200)	Invitrogen	Cat# A11001
Alexa 488 anti-rabbit secondary (1:200)	Invitrogen	Cat# A11008
Alexa 555 anti-rabbit secondary (1:200)	Invitrogen	Cat# A21428
Alexa 647 anti-guinea pig secondary (1:200)	Invitrogen	Cat# A21450
Alexa 647 anti-rabbit secondary (1:200)	Invitrogen	Cat# A21244

ESM Table 2. Real-time PCR Primer Sets.

Gene	Forward Primer Sequence	Reverse Primer Sequence
Aldh1a3	AGGCTGTATTAAGACCTTCAG	GGAAGTTCCATGGTGTAATG
Arx	TTCCAGAAGACGCACTACCC	CTGTCAAAGTGTGCCATCTCGTC
Atf3	GCTGCCAAGTGTCGAAACAAG	CAGTTTTCCAATGGCTTCAGG
Atf4	ATCCAGCAAAGCCCCACAAC	CAAGCCATCATCCATAGCCG
Beta2 (Neurod1)	ACTCCAAGACCCAGAAACTGTC	ACTGGTAGGAGTAGGGATGCAC
Bip (Hspa5)	AGGACAAGAAGGAGGATGTGGG	ACCGAAGGGTCATTCCAAGTG
Chop (Ddit3)	TTCACTACTCTTGACCCTGCGTC	CACTGACCACTCTGTTTCCGTTTC
Cyclophilin A	TGTGCCAGGGTGGTGACTTTAC	TGGGAACCGTTTGTGTTTGG
Edem1	GCAATGAAGGAGAAGGAGACCC	TAGAAGGCGTGTAGGCAGATGG
Erp72 (Pdia4)	AGTCAAGGTGGTGGTGGGAAAG	TGGGAGCAAAATAGATGGTAGGG
F4/80 (Adgre1)	CCTGGACGAATCCTGTGAAG	GGTGGGACCACAGAGAGTTG
Fkbp11	ACACGCTCCACATACACTACACGG	ATGACTGCTCTTCGCTTCTCTCCC
Foxo1	TTCAATTCGCCACAATCTGTCC	GGGTGATTTTCCGCTCTTGC
Gcg	CGTAAAAAGCAGATGAGCAAAGTG	GAACAGGTGTAGACAGAGGGAGTCC
Gck	CATTGAATCAGAGGAGGGCAGC	TAGTGGACTGGGAGCATTTGTGGG
Gipr	GCGTGCTCTACTGCTTCATCAAC	AACTTTCCAAGACCTCATCCCC
Glp1r	GGGTCTCTGGCTACATAAGGACAAC	AAGGATGGCTGAAGCGATGAC
Glut2 (Slc2a2)	CATTCTTTGGTGGGTGGC	CCTGAGTGTGTTTGGAGCG
Gpd2 (mGPDH)	AAAGACTGGAGCCCCACACTCTAC	ATCCCGTATTTCACCTCTGCTTC
Gpr40 (Ffar1)	TATTCCTGGGGTGTGTGTGTGG	CCAAGGGCAGAAAGAAGAGCAG
Gpx1	ACAGTCCACCGTGTATGCCTTC	CTCTTCATTCTTGCCATTCTCCTG
Hmox1	CCACACAGCACTATGTAAAGCGTC	GTTCGGGAAGGTAAAAAAAGCC
Il1b	TGTTCTTTGAAGTTGACGGACCC	CCACAGCCACAATGAGTGATACTG
Il6	CAAGAGACTTCCATCCAGTTGCC	CATTTCCACGATTTCCCAGAGAAC
Insulin	TCTTCTACACACCCATGTCCC	GGTGCAGCACTGATCTAC
Irx2	ACGCACACCACCGGAATG	ATGGATAGGCCGCACTGC
Kir6.2 (Kcnj11)	TCGTGTCCAAGAAAGGCAACTG	GGAAGGCAGATGAAAAGGAGTGG
Myc	GTCTTCCCCTACCCGCTC	CTGTCCAACTTGGCCCTC
Nkx6.1	GGACCAGAGAGAGCACGC	TTCGGGTCCAGAGGTTTG
p21 (Cdkn1a)	CTTGTCGCTGTCTTGCACTCTG	CTTCAGGGTTTTCTCTTGCAGAAG
p53 (Trp53)	ACTGCATGGACGATCTGTTG	GTGACAGGGTCCTGTGCTG
Pcx	GTTCCGTGTCCGAGGTGTAAAG	CGCAGAAGGATGTCCCTGAAAC
Pc2 (Pcsk2)	GCATACAACTCCAAGGTGGCAG	CCATCAACCGTCTTCCCATTG
Pdx1	CGGACATCTCCCCATACG	AAAGGGAGCTGGACGCGG

Sod1	ATGGGGACAATACACAAGGCTG	CAATGATGGAATGCTCTCCTGAG
Sox9	CGACTACGCTGACCATCAGA	AGACTGGTTGTTCCCAGTGC
Tnf	CCCCTTTACTCTGACCCCTTTATTG	AACCTGACCACTCTCCCTTTGC
Trib3	TCTTCAGCAACTGTGAGAGGACG	TCCAGACATCAGCCGCTTTG
Xbp1	GCAGCAAGTGGTGGATTTGG	AGATGTTCTGGGGAGGTGACAAC
Xbp1s	GAGTCCGCAGCAGGTG	GTGTCAGAGTCCATGGGA

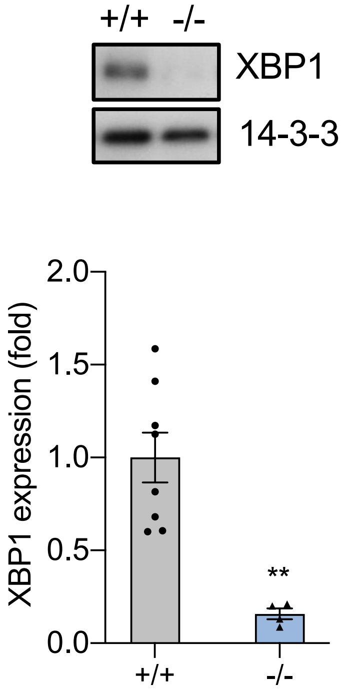

ESM Fig. 1. XBP1 protein levels in islets of β-Xbp1$^{+/+}$ and β-Xbp1$^{-/-}$ mice fed a chow diet. Islets were isolated and protein analyzed by immunoblotting. Bands for XBP1 were quantified by densitometry and normalized to 14-3-3. All data are represented as means ± SEM expressed as fold change of the levels in β-Xbp1$^{+/+}$ mice. n = 8, β-Xbp1$^{+/+}$; n = 4, β-Xbp1$^{-/-}$. Unpaired two-tailed *t*-test: $^{**}p<0.01$.

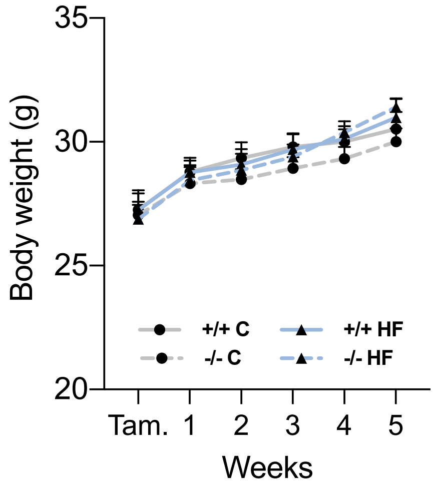

ESM Fig. 2. Body weight of β-Xbp1$^{+/+}$ and β-Xbp1$^{-/-}$ mice fed a chow or a high-fat diet. All data are represented as means ± SEM. n = 9, Chow-fed β-Xbp1$^{+/+}$; n = 13, High-fat-fed β-Xbp1$^{+/+}$; n = 8, Chow-fed β-Xbp1$^{-/-}$; n = 13, High-fat-fed β-Xbp1$^{-/-}$. Chow-fed, C; High-fat-fed, HF.

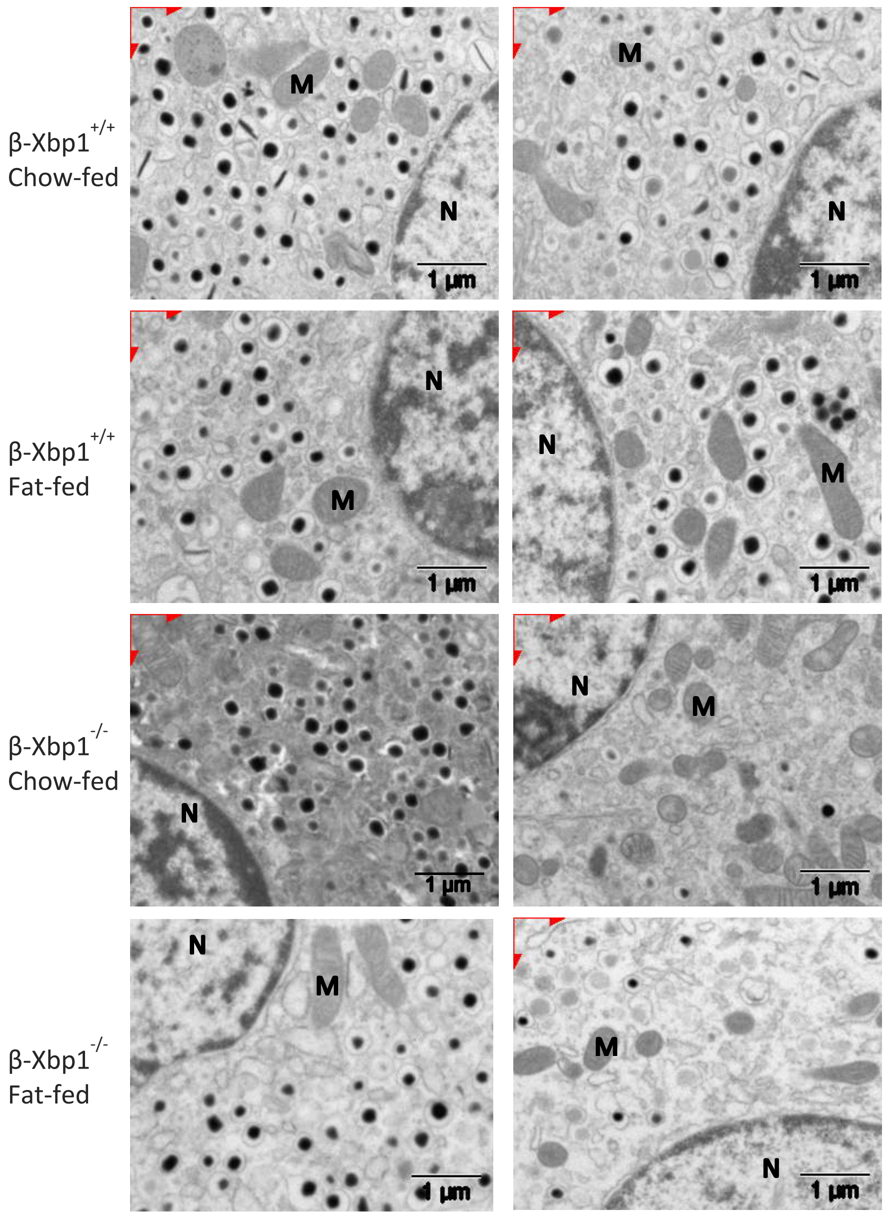

ESM Fig. 3. Ultrastructure analysis of isolated islets by transmission electron microscopy. M, mitochondria. N, nucleus. Magnification bar, 1 µm. Representative images are shown from chow-fed β-Xbp1$^{+/+}$ (n = 5), high-fat-fed β-Xbp1$^{+/+}$ (n = 7), chow-fed β-Xbp1$^{-/-}$ (n = 6) and high-fat-fed β-Xbp1$^{-/-}$ (n = 5) mice.

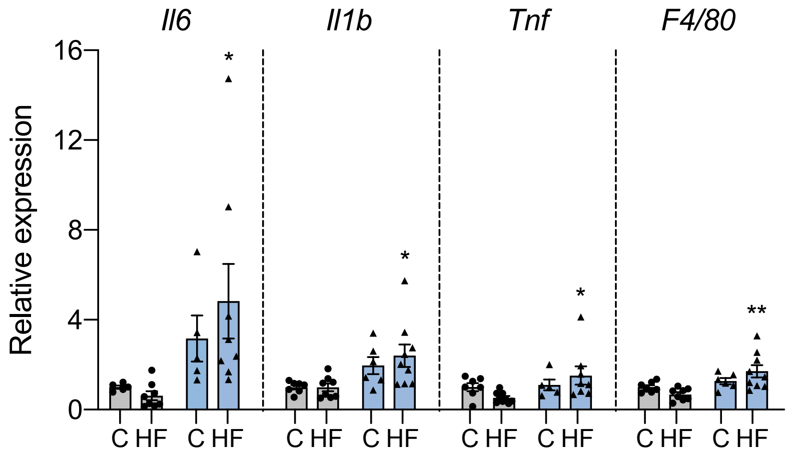

ESM Fig. 4. Changes in mRNA expression of inflammation genes (cytokines, *Il6*, *Il1b* and *Tnf* and the macrophage marker, *F4/80*) in islets of β-Xbp1$^{+/+}$ and β-Xbp1$^{-/-}$ mice fed a chow or high-fat diet. Islets were isolated and RNA was extracted, reverse-transcribed and analyzed by real-time RT-PCR. All data are represented as means ± SEM expressed as fold change of the levels in chow-fed β-Xbp1$^{+/+}$ mice. n = 5-7, Chow-fed β-Xbp1$^{+/+}$; n = 8, High-fat-fed β-Xbp1$^{+/+}$; n = 5-6, Chow-fed β-Xbp1$^{-/-}$; n = 8-9, High-fat-fed β-Xbp1$^{-/-}$. ANOVA: **p*<0.05, ***p*<0.01 genotype effect. Chow-fed, C; High-fat-fed, HF.

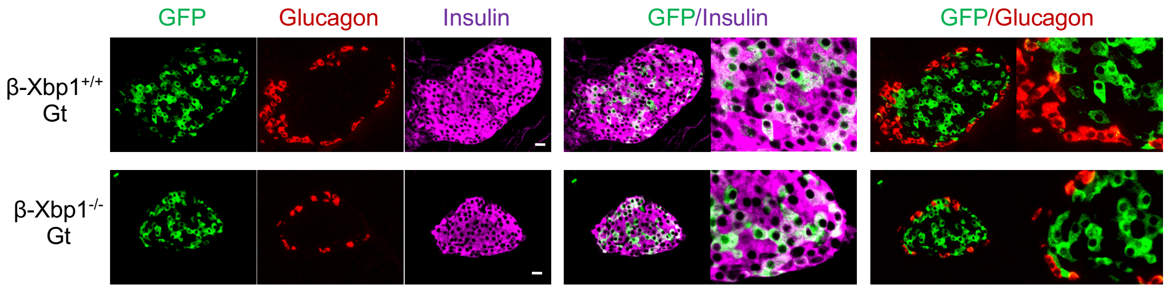

ESM Fig. 5. Baseline labelling pattern for cell-type-specific lineage tracing in β-Xbp1$^{+/+}$Gt and β-Xbp1$^{-/-}$Gt mice. One day after mice received the final i.p. tamoxifen injection, pancreas was collected and sections immunostained for GFP (green), glucagon (red) and insulin (magenta). Representative images of immunostaining are shown. Scale bar, 20 µm. n = 5, β-Xbp1$^{+/+}$Gt; n = 6, β-Xbp1$^{-/-}$Gt

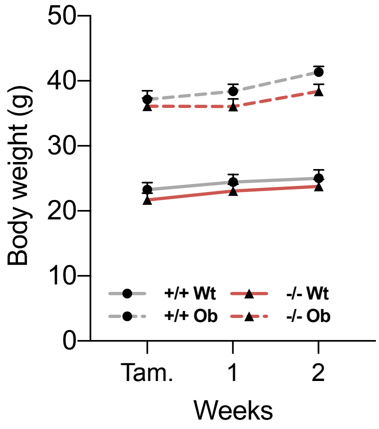

ESM Fig. 6. Body weight of β-Xbp1$^{+/+}$Wt (n = 9), β-Xbp1$^{-/-}$Wt (n = 13), β-Xbp1$^{+/+}$Ob (n = 5) and β-Xbp1$^{-/-}$Ob (n = 10) mice. All data are represented as means ± SEM.

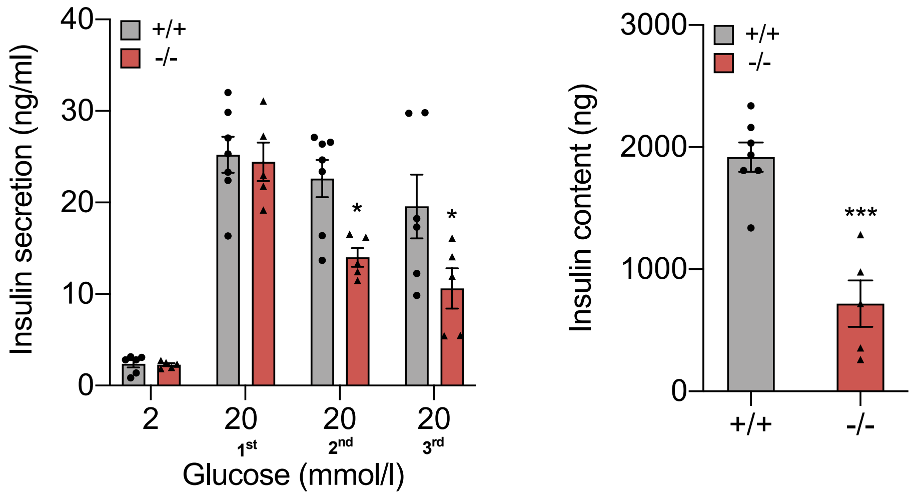

ESM Fig. 7. Insulin secretion and insulin content in islets of β-Xbp1$^{+/+}$Wt and β-Xbp1$^{-/-}$Wt mice treated with repeated low (2 mmol/l) or high (20 mmol/l) stimulatory level of glucose for three cycles. n = 7, β-Xbp1$^{+/+}$Wt; n = 5, β-Xbp1$^{-/-}$Wt. *p<0.05, ***p<0.001.

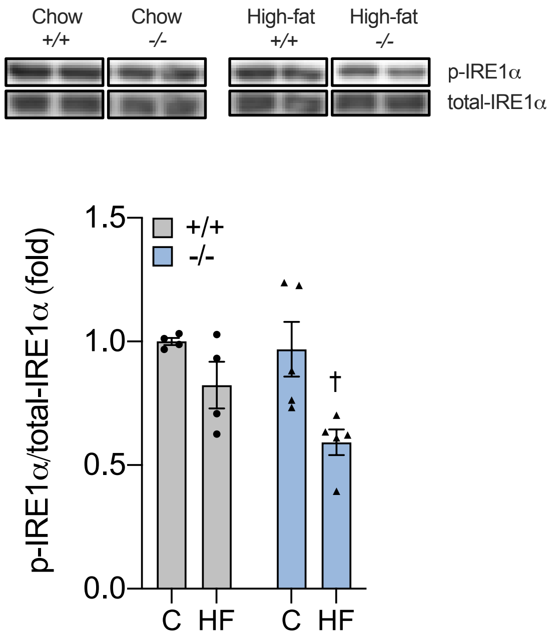

ESM Fig. 8. IRE1α phosphorylation in islets of β-Xbp1$^{+/+}$ and β-Xbp1$^{-/-}$ mice fed a chow or high-fat diet. Islets were isolated and protein analyzed by Western blotting. Bands for phosphorylated (p)-IRE1α were quantified by densitometry and normalized to total IRE1α. All data are represented as means ± SEM expressed as fold change of the levels in chow-fed β-Xbp1$^{+/+}$ mice. n = 4, Chow-fed β-Xbp1$^{+/+}$; n = 4, High-fat-fed β-Xbp1$^{+/+}$; n = 5, Chow-fed β-Xbp1$^{-/-}$; n = 5, High-fat-fed β-Xbp1$^{-/-}$. ANOVA: $^{\dagger}p<0.01$, diet effect. Chow-fed, C; High-fat-fed, HF.

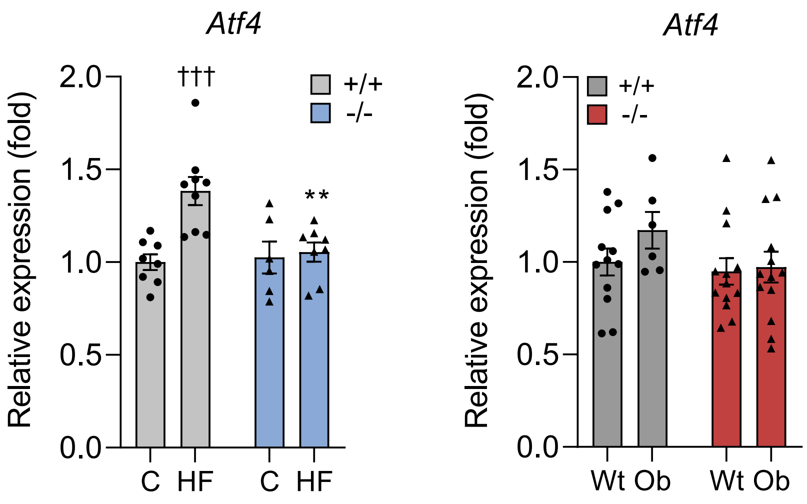

ESM Fig. 9. Changes in *Atf4* mRNA expression in islets of mice. Islets were isolated and RNA was extracted, reverse-transcribed and analyzed by real-time RT-PCR. All data are represented as means ± SEM expressed as fold change of the levels in respective control mice. (A) n = 8, Chow-fed β-Xbp1$^{+/+}$; n = 9, High-fat-fed β-Xbp1$^{+/+}$; n = 6, Chow-fed β-Xbp1$^{-/-}$; n = 8, High-fat-fed β-Xbp1$^{-/-}$. ANOVA: $^{**}p<0.01$ genotype effect. $^{†††}p<0.001$ diet effect. Chow-fed, C; High-fat-fed, HF.

(B) n = 12, β-Xbp1$^{+/+}$Wt; n = 6, β-Xbp1$^{-/-}$Wt; n = 13, β-Xbp1$^{+/+}$Ob; n = 13, β-Xbp1$^{-/-}$Ob.
